# QuickStats

**Published:** 2013-11-15

**Authors:** Yechiam Ostchega, Steven M. Frenk

**Figure:**
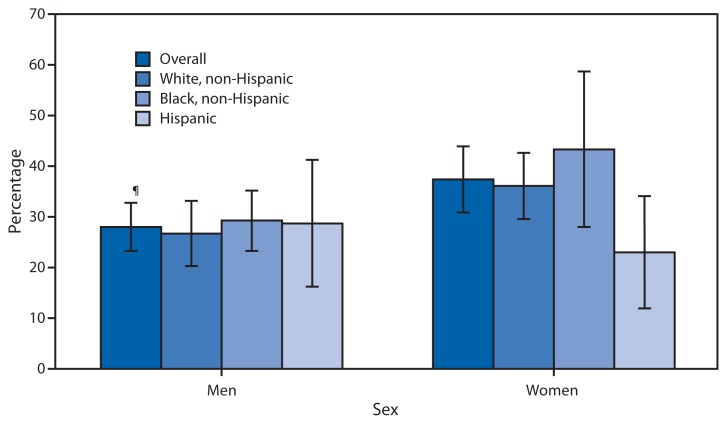
Percentage of Adults with Hypertension* Who Monitored Their Blood Pressure at Home at Least Once a Month,^†^ by Sex and Race/Ethnicity — National Health and Nutrition Examination Survey, United States, 2009–2010^§^ * Hypertension was defined as systolic blood pressure ≥140 mm Hg, diastolic blood pressure ≥90 mm Hg, or currently taking medication to lower blood pressure, based on affirmative responses to the following questions: “Have you ever been told by a doctor or other health professional that you had hypertension, also called high blood pressure?”; “Because of your [high blood pressure/hypertension], have you ever been told to take prescribed medicine?”; and “Are you now taking a prescribed medicine?” ^†^ Data on home blood pressure monitoring come from two questions. Respondents were first asked, “Did you take your blood pressure at home during the last 12 months?” Respondents who answered “yes” were then asked, “How often did you check your blood pressure at home during the last 12 months?” ^§^ All estimates are age-adjusted to the 2000 projected U.S. standard population using the age groups 18–39, 40–59, and ≥60 years. ^¶^ 95% confidence interval.

During 2009–2010, approximately 32% of adults aged ≥18 years with hypertension reported that they monitored their blood pressure at home at least once a month. Women with hypertension were more likely to monitor their blood pressure than men with hypertension (37% versus 28%). Non-Hispanic black women with hypertension were more likely to monitor their blood pressure at home than Hispanic women with hypertension. No differences were observed by race or Hispanic ethnicity among men.

**Source:** Ostchega Y, Berman L, Hughes JP, Chen TC, Chiappa MM. Home blood pressure monitoring and hypertension status among US adults: the National Health and Nutrition Examination Survey (NHANES), 2009–2010. Am J Hypertens 2013;26:1086–92.

